# The Role of Fibroblast Growth Factor 19 Subfamily in Different Populations Suffering From Osteoporosis

**DOI:** 10.3389/fendo.2022.830022

**Published:** 2022-04-28

**Authors:** Xiawen Yu, Yue Xia, Jue Jia, Guoyue Yuan

**Affiliations:** Department of Endocrinology and Metabolism, The Affiliated Hospital of Jiangsu University, Zhenjiang, China

**Keywords:** myokine, fibroblast growth factor 19 subfamily, FGF21, osteoporosis, metabolic syndrome, polycystic ovary syndrome

## Abstract

Fibroblast growth factor (FGF) 19 subfamily, also known as endocrine fibroblast growth factors (FGFs), is a newly discovered metabolic regulator, including FGF19, FGF21 and FGF23. They play significant roles in maintaining systemic homeostasis, regulating the balance of bile acid and glucolipid metabolism in humans. Osteoporosis is a chronic disease, especially in the current status of aging population, osteoporosis is the most prominent chronic bone disease, leading to multiple complications and a significant economic burden that requires long-term or even lifelong management. Members of the FGF family have been shown to be associated with bone mineral density (BMD), fracture repair and cartilage regeneration. Studies of the FGF19 subfamily in different populations with osteoporosis have been increasing in recent years. This review summarizes the role of the FGF19 subfamily in bone metabolism, and provides new options for the treatment of bone diseases such as osteoporosis.

## Introduction

Osteoporosis is a systemic skeletal disease caused by disorders of bone metabolism. An article published in Lancet in 2019 showed that fractures resulting from osteoporosis became increasingly common in women after age 55 years and men after age 65 years, leading to increased substantial bone-associated morbidities, mortality and health-care costs (1). It can be seen that osteoporosis is one of the main causes of disability and death in the elderly (2, 3). Therefore, the incidence and complications of osteoporosis should arouse public attention.

Earlier studies have suggested that osteoporosis is likely to be caused by complex interactions among local and systemic regulators of bone cell function (4). Recent studies have found that skeletal muscle, acting as an endocrine organ, can produce a variety of important myokines, which are associated with the pathogenesis of osteoporosis (5). As a myokine, the fibroblast growth factor (FGF) family plays an important role in tissue homeostasis, repair, regeneration, angiogenesis and bone metabolism (6). Adhikary et al. treated primary osteoblasts and C2C12 myoblasts with fibroblast growth factor-2 (FGF-2) and dexamethasone, and found that exogenous FGF-2 alleviated the GC induced effects by inhibiting the expression of sclerostin and myostatin in bone and muscle respectively (7). Therefore, they believed that exogenous FGF-2 can maintain osteogenesis and inhibit muscular atrophy in the presence of GC, suggesting that FGF-2 may be a potential target for the treatment of osteoporosis. Activating mutations in fibroblast growth factor receptor 2 (FGFR2) cause several craniosynostosis syndromes by affecting the proliferation and differentiation of osteoblasts, which form the calvarial bones (8). In addition, FGF-2 was involved in fracture repair, bone formation and cartilage regeneration after fracture damage or strenuous exercise (9). In mammals, the FGF family has 23 members (including FGF-15 in mice) and consists of 22 peptides (10). The FGF19 subfamily, also known as endocrine fibroblast growth factors (FGFs), consisting of FGF-19, FGF-21 and FGF-23, has been a hot topic of research in recent years (10). They bind to FGF receptors (FGFRs) through blood circulation and play a regulatory role in phosphate, bile acid, carbohydrate and lipid metabolism (11). Due to endocrine FGFs have a low affinity for FGFRs, they can freely cross the HS-dense cell gap into the bloodstream and eventually form complexes with Klotho proteins to stimulate cellular activity (12, 13). Endocrine FGFs are involved in the metabolic activities of various organs such as parathyroid glands, kidney, liver and adipose tissue through the binding of Klotho proteins to the corresponding FGFRs (14–18). At present, several members of the FGF family have been shown to be involved in the regulation of skeletal muscle growth and development, and studies on the endocrine regulation of the FGF19 subfamily in different populations with osteoporosis have been increasing in recent years. In this review, we will focus on recent findings on the association of the FGF19 subfamily with osteoporosis and its role in different populations suffering from osteoporosis.

## The Endocrine Subfamily of FGFs

### FGF-19

FGF-19 is secreted by ileal epithelial cells and plays a regulatory role in the maintenance of bile acid and corresponding metabolic homeostasis (19). Once secreted, FGF-19 triggers a signaling cascade involving the recruitment of cytosolic articulators by binding to its preferred receptor FGFR4 and co-receptor β-klotho (**Table 1**). Although FGF-19 is primarily metabolized in the liver through activation of the FGFR4-β-klotho complex, studies in recent years have shown that FGF-19 also performs biological functions in white adipose tissue (WAT) and brain (11). In addition, studies have found that the FGF19 levels are significantly lower in postmenopausal patients with osteoporosis than in healthy women and are positively correlated with bone mineral density (BMD) (20).

**Table 1:** Endocrine FGF physiological function.

Endocrine FGFs	Receptor complex	Functions
FGF19	FGFR4-β-klotho	↓Bile acid synthesis ↓Triglycerides ↓Gluconeogenesis ↑Glycogen and protein synthesis
FGF21	FGFR1c-β-klotho	↑Hepatic fatty acid oxidation ↑Gluconeogenesis ↑WAT browing ↑weight loss ↑Glucose absorption ↑Insulin sensitivity
FGF23	FGFR1c-α-klotho FGFR3c-α-klotho FGFR4-α-klotho	↓Renal phosphate absorption ↓Vitamin D synthesis ↓PTH secretion

### FGF-21

FGF-21 plays a key role in the metabolic process as a hepatic, adipokine and myokine. It appears to function through FGFR1c-mediated binding to β-klotho to form complexes (21). Circulating FGF21 is primarily expressed by the liver when the body is starving, obese, mitochondrial dysfunction and aging, while FGF21 expression is barely detectable in healthy conditions (22). In recent years, numerous studies have shown that increasing the level of myogenic FGF-21 could enhance skeletal muscle glucose uptake, fatty acid oxidation and insulin sensitivity, thereby improving lipid metabolism and reducing body weight (23–25). In addition, FGF-21 has also been shown to be involved in the browning of white fat (26). Up to now, the research on the relationship between FGF21 and BMD has inconsistent results in animals or humans, possibly related to the different experimental subjects. In addition, recombinant human FGF21 (rhFGF21) injected into mice may undergo biochemical reactions (27–29).

### FGF-23

**Figure 1:**
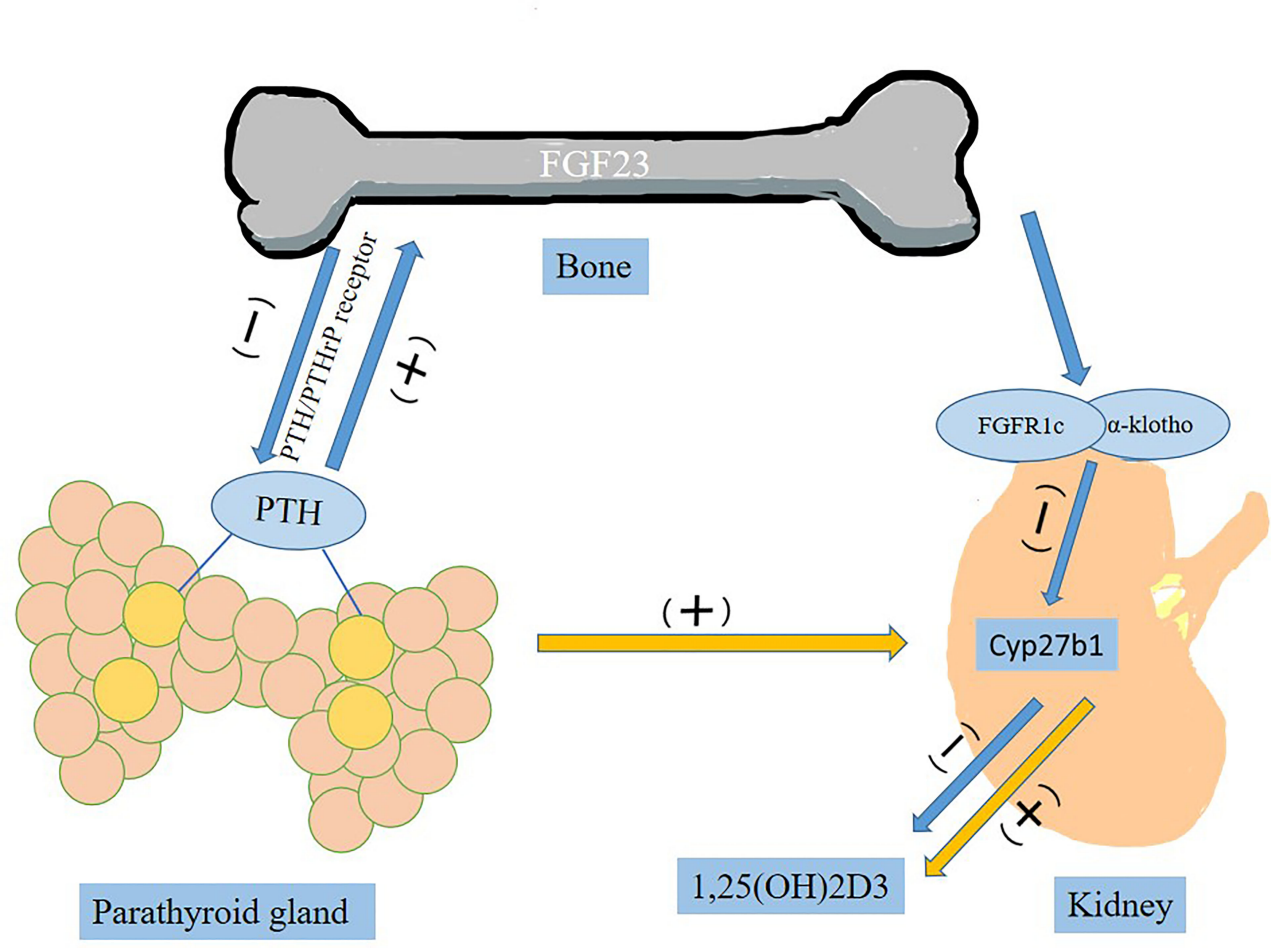
The role of FGF23 in the circulation.

FGF-23 is known as a bone-derived endocrine hormone, mainly secreted by osteocytes and osteoblasts, mediated by the FGFR (FGFR1c, FGFR3c and FGFR4) and combined with α-Klotho to stimulate phosphate excretion and inhibit formation of 1, 25(OH)2D3, active vitamin D (11). Parathyroid hormone (PTH) stimulates synthesis and secretion of FGF23 by activating PTH/PTHrP receptor on osteocytes/osteoblasts (**Figure 1**) (30). In the parathyroid glands, FGF-23 is involved in metabolic activities by downregulating the production and secretion of PTH (31). It suppresses the synthesis of 1,25(OH)2D3 by inhibiting key enzyme 1-α-hydroxylase (encoded by Cyp27b1) in the kidney (31). In complete contrast to FGF23, PTH acts on the kidney to upregulate Cyp27b1 expression and increase blood 1,25(OH)2D3 levels (32).

## FGF19 Subfamily in Metabolic Syndrome (MetS)

MetS is a global health problem increasing worldwide, which includes a set of common metabolic abnormalities such as insulin resistance, impaired glucose tolerance, abdominal obesity, dyslipidemia, and hypertension (33). The interaction of these factors makes the body susceptible to cardiovascular disease, diabetes and osteoporosis (34). Wong et al. fed rats with a high-carbohydrate high-fat (HCHF) diet with 25% fructose-supplemented drinking water to induce MetS (35). They detected elevated levels of FGF-23 in bone after MetS establishment, which subsequently led to significant bone loss, with alterations of bone histomorphometric parameters characterized by increased osteoclastic activity and decreased osteoblastic activity (35). In humans, the positive relationship between MetS and BMD was driven by high BMI (36, 37), but when fat mass was considered alone, MetS had a negative effect on BMD (37). In addition, gender was an interesting factor in the relationship, the negative effects of MetS were more prominent in men compared to women (37). Bone formation and resorption are mainly controlled by osteoblasts and osteoclasts, and imbalance in this process leads to deterioration of bone microstructure and bone strength, ultimately promoting the development of osteoporosis.

Vitamin D is an important factor in bone metabolism. Animal studies have shown that vitamin D3 supplementation has a positive effect on fracture healing (38). A clinical study found that serum FGF19 levels were negatively correlated with body weight and hip BMD in older adults older than 60 years (39). Furthermore, FGF-19 was associated with treatment of active vitamin D3, because all patients taking active vitamin D3 drugs had significantly higher serum FGF19 levels than controls and tended to show improvements in serum parameters related to lipid metabolism, such as high high-density lipoprotein (HDL) and low triglyceride (TG) levels (39). Since MetS is prone to osteoporosis, the improvement of blood lipids and pressure is beneficial to reduce the prevalence of osteoporosis in MetS patients. What’s more, this experiment also found that FGF-21 was associated with TG levels, and hypertension. The serum FGF21 levels also tended to be high in patients with dyslipidemia (39). At the same time, they also found that FGF-23 was negatively associated with markers for bone formation and absorption, namely tartrate-resistant acid phosphatase 5b (TRACP5b), suggesting that high FGF23 levels may be associated with low bone turnover (39). TRACP5b can be used as a diagnostic and therapeutic monitor for early osteoporosis (40). Finally, this article mentioned that measurement of FGF-21 and FGF-23 was useful for detecting chronic kidney disease (CKD) and its complications, such as cardiovascular disease and metabolic bone disease (39). In conclusion, FGF-21 and FGF-23, as members of the FGF subfamily, are involved in MetS and bone metabolic activities and may subsequently provide new options for the treatment of osteoporosis and MetS. FGF-19 is associated with the pharmacological treatment of active vitamin D3 and may improve MetS by improving serum lipid metabolism-related parameters such as HDL and TG in patients taking active vitamin D3 drugs.

The decline of renal function in CKD is accompanied by chronic kidney disease-mineral and bone disorder (CKD-MBD), which can lead to renal osteodystrophy and osteoporosis (41). When kidney function is severely impaired, kidney transplantation (KT) is the treatment of choice for most patients with renal failure. However, the early period after KT (the first 6 months) is a period of high risk for major fractures (MF), and the incidence of fractures is higher in women than in men (42). A retrospective study of 74 KT patients found that higher FGF-23 levels and lower HDL cholesterol in KT with MetS compared to controls (43). Therefore, they concluded that high serum FGF-23 levels were positively associated with MetS in KT patients. In addition, they used multivariable logistic regression analysis found that FGF-23 was an independent predictor of MetS in KT patients (43).

## FGF19 Subfamily in Postmenopausal Osteoporosis (PMO)

The risk of bone fracture increases with age, especially in postmenopausal women, who have the highest prevalence of osteoporosis of all bone metabolic diseases, so women should be screened for osteoporosis starting at age 65 (44, 45). As the physiological process progresses in menopausal women, there is a significant decrease in estrogen secretion, an increase in osteoclast activity, and a decrease in bone matrix secretion (46). It’s worth noting that bone formation actually increases after menopause, but resorption increases even more. As a result, the body is unable to effectively regulate bone resorption and bone formation, resulting in a decrease in bone mass and bone density, which ultimately leads to osteoporosis.

Previous studies have shown that FGF21 enhanced PPAR-γ activity by inhibiting osteoblastogenesis and increasing bone marrow adipogenesis in bone marrow mesenchymal stem cells (BMSCs) to increase bone resorption and decrease bone formation (47). Recombinant FGF21 protein improved insulin sensitivity, lowered blood glucose and TG, and reduced body weight in diabetic mice (48). However, it was reported that high fat diet-induced obesity (DIO) mice given continuous 4-week intraperitoneal injections of rhFGF21 found no effect of rhFGF21 on bone mass or any bone biomarkers. Li et al. also administered rhFGF21 intraperitoneally to DIO mice for 2 weeks and observed the same results (49). In addition, they intervened with the PPAR-γ agonist rosiglitazone in wild-type (WT) and FGF21 knockout (KO) mice and found that increased adipogenesis and bone marrow adipocytes in rosiglitazone intervened mice, but not in FGF21 intervened mice (49). Thus, they concluded that FGF21 does not appear to be a downstream mediator of PPAR-γ on adipocyte differentiation, nor does it play a role in rosiglitazone-induced bone loss, and that the pathway of FGF21 and PPAR-γ appears to be independent (49).

However, Studies on the relationship between FGF21 levels and BMD have yielded inconsistent results in humans. A cross-sectional analysis of healthy postmenopausal women showed that their circulating FGF-21 levels were positively correlated with lumbar spine BMD (27). One study showed no correlation between FGF21 levels and BMD (28). Another study revealed that plasma FGF21 levels were inversely correlated with BMD in femoral neck and Ward’s triangle of hip region (29). This inconsistent may be due to the different experimental subjects. Li’s subjects were DIO mice, while the other study subjects were PMO patients (postmenopausal Han women). In addition, Li et al. injected rhFGF21 into DIO mice, and the protein may undergo metabolic activity in the mice.

A cross-sectional study of 28 patients with PMO, 32 with postmenopausal osteopenia and 30 healthy control subjects (postmenopausal non-osteoporosis) found that significantly higher levels of FGF-23 in the PMO group compared to the postmenopausal osteopenia and control groups (50). In addition, PMO patients had significantly lower levels of lomber and femur BMD than postmenopausal osteopenia and control groups. When subjects in the PMO group were divided into three groups according to age of menopause, the FGF-23 levels were found to be significantly higher in the group of menopausal age <5 years than the group of menopausal age >10 and the group of menopausal age 5-10 years. Therefore, they concluded that serum FGF-23 level was an important determinant of increased bone turnover at early periods in PMO patients (50). It can be seen that both FGF21 and FGF23 are involved in bone metabolic processes in PMO patients and have an impact on BMD. A prospective study of PMO found that the anabolic effect of PTH on osteoblasts led to an increase in FGF-23 when PTH was given intermittently (51). Therefore, this study suggested that FGF-23 may mediate the skeletal anabolic effects of parathyroid hormone (51).

Zhao et al. divided 150 postmenopausal Chinese women into osteoporosis group, osteopenia group, and healthy control group based on their BMD, and assessed serum bile acid, FGF19, and bone turnover biomarker levels (20). This cross-sectional study found that serum bile acid and FGF19 levels were significantly lower in PMO and osteopenia than in healthy women. In addition, serum total bile acid and FGF19 levels were positively correlated with BMD (20). This finding also suggested that bile acid played an important role in bone metabolism based on clinical evidence (20). FGF19 is the downstream molecule of bile acid signaling. Bile acid binds to and activates farnesoid X receptor (FXR) in the small intestinal cells to induce the upregulation of FGF19 (52). FXR is expressed not only in the liver and intestine, which are the classical target organs of bile acids, but also in bone marrow stromal cells and SaOS2 osteoblast-like cells (52). One study found that *in vivo* deletion of FXR resulted in a significant reduction in bone mass in mice (53). FGF15/19, as an intestine-derived endocrine hormone, plays a key role in mediating the gut- hepatic bile acid signalling feedback to inhibit hepatic bile acid synthesis (54). Therefore, bile acids can up-regulate FGF19 by activating FXR to participate in bone metabolism. Studies have found that bile acid-induced FGF19 acts through mTOR/ERK signaling and transcriptional factor EB (TFEB) phosphorylation to feedback inhibit TFEB nuclear translocation in hepatocytes (55). Wang et al. revealed that TFEB induced cholesterol 7α-hydroxylase (CYP7A1) in human hepatocytes and mouse livers, and prevented hepatic cholesterol accumulation and hypercholesterolemia in Western diet mice-fed mice in a high fat, high sucrose, high cholesterol manner (55). A recent study found that administration of SH-479, which is a bile acid receptor agonist, to mice with PMO increased BMD and improved skeletal microarchitecture (56). Therefore, bile acids were considered to be used in the treatment of metabolic diseases, such as type 2 diabetes, hyperlipidemia, and obesity (57, 58). In conclusion, the bile acid metabolic pathway involved in FGF19 could be a new therapeutic target for PMO (20).

## FGF19 Subfamily in Polycystic Ovary Syndrome (PCOS)

PCOS is the most common endocrine metabolic disorder in women of reproductive age and is defined by a combination of signs and symptoms of androgen excess and ovarian dysfunction (59). It is often associated with abdominal obesity, insulin resistance, obesity, metabolic disorders, and cardiovascular risk factors (60). In addition, women with PCOS are at higher risk of insulin resistance, hypertension, dyslipidemia, diabetes and osteoporosis (61). Studies have shown that hyperinsulinemia may play a crucial role in the development of PCOS and lead to elevated androgen levels, yet increased androgens in women with PCOS can lead to insulin resistance (61). In addition, insulin may stimulate osteoblast differentiation, thereby enhancing osteocalcin production (62). Osteocalcin, a peptide produced and secreted by osteoblasts, is associated with bone synthesis and conversion, and stimulates pancreatic β cell proliferation and skeletal muscle insulin sensitivity (63). Elevated insulin levels in women with PCOS lead to insulin resistance, which in turn leads to the deterioration of BMD (61). In addition, the ovarian and adrenal-derived hyperandrogenemia in women with PCOS could affect bone turnover and BMD (64). Therefore, we explored the findings of studies related to FGFs and PCOS.

FGFs not only play a key role in development, cell growth, tissue repair and transformation, but also stimulate the ovarian granulosa cell differentiation, expression of the luteinizing hormone (LH) receptors by granulosa cells, and proliferation of ovarian germinal cells (65). Among the fibroblast growth factor family, FGF-13 and FGF-18 are associated with ovarian function (66). FGF-21 is mainly expressed by the liver and acts as a potent activator of glucose uptake by inducing glucose transporter 1 (GLUT1) on adipocytes, which reverses hepatic steatosis and improves insulin sensitivity in obese mice (67). A study on the effect of insulin on endocrine FGFs in women with PCOS found that insulin administration increased plasma levels of FGF-21 in healthy controls and women with PCOS, suppressed plasma levels of FGF-19 in healthy controls, and had no effect on plasma levels of FGF-23 (21). An earlier study evaluated FGF-21 levels in PCOS and found that circulating FGF-21 levels were higher in PCOS patients and correlated with homeostasis model assessment insulin resistance index (HOMA-IR) (68). However, Sahin et al. found that serum concentrations of FGF-21 were not different in PCOS patients compared to the healthy group (67). Furthermore, FGF-21 levels did not correlate with metabolic parameters such as BMI, fasting glucose, insulin, HOMA-IR and lipid parameters in PCOS patients. Therefore, they concluded that FGF-21 was not a useful marker for metabolic abnormalities such as insulin resistance, dyslipidemia, obesity and hypertension in women with PCOS (67). Subsequently, it has also been discovered that circulating FGF21 levels were associated with obesity but not with PCOS (69). The reason for these two different experimental results may be that the earlier study involved only a small sample of 24 PCOS patients and 13 healthy controls, whereas the latter two studies included a larger number of study subjects. In addition, the different ethnicity of the study subjects may also have influenced the results. Certainly, there are few studies on the relationship between PCOS and circulating FGF21 levels, and more studies are needed to further elucidate the role of FGF-21 in glucose homeostasis, especially in patients with PCOS.

## Conclusions

Overall, the FGF19 subfamily has been the focus of attention since its discovery. A large number of studies in recent years have revealed that the FGF19 subfamily is associated with bone metabolism, which has caused controversies and debates. Serum FGF19 and bile acid levels are positively correlated with BMD, while both levels are reduced in the serum of PMO patients. Therefore, FGF19 may provide a new therapeutic target for PMO patients by participating in the enterohepatic circulation of bile acid. The relationship between FGF21 and BMD is controversial in both mouse and human studies, but FGF21 does participate in the metabolic activity of MetS, PMO and POCS patients, and more basic and clinical experiments are needed to further clarify the relationship subsequently. As a bone-derived hormone, FGF23 can act on kidneys and parathyroid glands to regulate bone metabolism activity, which may offer new options for the treatment of osteoporosis and other bone diseases. In addition, scientific consensus has unanimously established that the FGF19 subfamily, especially FGF21, is a new key player in bone metabolism and its role is emerging as a possible therapeutic option to treat bone diseases.

## Author Contributions

JJ and GY designed the study. XY wrote the paper. XY and YX made the figure and table. JJ and GY revised the paper. All authors read and approved the final manuscript.

## Funding

This study was supported by the National Natural Science Foundation of China (81870548, 81570721, 81500351), the Social Development Project of Jiangsu Province (BE2018692), the Natural Science Foundation of Jiangsu Province, China (BK20191222), the Youth Medical Talent Project of Jiangsu Province (QNRC2016842), the High Caliber Medical Personnel Foundation of Jiangsu Province (LGY2016053), the Six Talent Peaks Project in Jiangsu Province (2015-WSN-006), the Jiangsu University Affiliated Hospital “5123” Talent Plan (51232017305), the sixth “169 “ Talent Project of Zhenjiang, the Science and Technology Commission of Zhenjiang City (FZ2020038), Doctoral Research Initiation Fund (jdfyRC2020010) and Clinical Medical Science and Technology Development Foundation of Jiangsu University (JLY2021209).

## Conflict of Interest

The authors declare that the research was conducted in the absence of any commercial or financial relationships that could be construed as a potential conflict of interest.

## Publisher’s Note

All claims expressed in this article are solely those of the authors and do not necessarily represent those of their affiliated organizations, or those of the publisher, the editors and the reviewers. Any product that may be evaluated in this article, or claim that may be made by its manufacturer, is not guaranteed or endorsed by the publisher.
